# Warfarin is associated with the risk of vascular calcification in abdominal aorta in hemodialysis patients: a multicenter case-control study

**DOI:** 10.3906/sag-2104-221

**Published:** 2021-10-21

**Authors:** Rezzan EREN SADİOĞLU, Evren ÜSTÜNER, İhsan ERGÜN, Süleyman Tevfik ECDER, Gökhan NERGİZOĞLU, Kenan KEVEN

**Affiliations:** 1 Division of Nephrology, Department of Internal Medicine, Faculty of Medicine, Ankara University, Ankara Turkey; 2 Department of Radiology, Faculty of Medicine, Ankara University, Ankara Turkey; 3 Division of Nephrology, Department of Internal Medicine, Faculty of Medicine, Ufuk University, Ankara Turkey; 4 Division of Nephrology, Department of Internal Medicine, Faculty of Medicine, Demiroğlu Bilim University, Istanbul Turkey

**Keywords:** Hemodialysis, vascular calcification, vitamin K, warfarin

## Abstract

**Background/aim:**

Vascular calcifications (VCs), recognized risk factor for increased mortality, are highly prevalent in hemodialysis (HD) patients. We aimed to investigate the relation between VC and warfarin use with plain radiography.

**Materials and methods:**

VCs were assessed using Adragao (radial and digital) and Kauppila (aortic) scores in 76 HD patients from six centers. Out of a total 711 HD patients, there were 32 (4.5%) who had been treated with warfarin for at least 1 year, and we included 44 control patients.

**Results:**

Of the patients, 47% were females, the mean age was 66 ± 9 years, 23% were diabetics, the mean dialysis vintage was 68 ± 38 months. In warfarin group, median Kauppila score was higher than in control group [11 vs 6.5, (25%–75% percentile, 5 vs. 15), p = 0.032] and the percentage of the patients with a Kauppila score of >6 was higher, as well (76.6% vs. 50%; p = 0.029). Median Adragao score was not significantly different between the two groups [7 vs. 6, (%25,%75 percentile 6 vs. 8), p = 0.17]. Logistic regression analysis revealed that warfarin treatment was independently associated with Kauppila scores of >6 (OR 3.60, 95% CI 1.18–10.9, p = 0.024).

**Conclusion:**

In this study, we found that warfarin is associated to vascular calcifications, especially in aorta of HD patients.

## 1. Introduction

Vascular calcification (VC) is a pathological mineralization process occuring mainly in elastic and muscular arteries such as aorta, iliac, coronary and peripheral arteries. VCs are highly prevalent and related to increased mortality in end-stage kidney diseases [1]. VC is an active cellular process and occurs as a result of the promoters for calcification overwhelming the inhibitors. Matrix Gla Protein (MGP), a molecule secreted by chondrocytes and vascular smooth muscle cells, is the most significant known natural calcification inhibitor, and it needs Vitamin K-dependent carboxylation and phosphorylation in order to be active [2]. Warfarin is an old drug that exerts its anticoagulant effects by blocking the γ-carboxylation of coagulation factors, resulting, therefore, in the inhibition of the activation of other vitamin K-dependent proteins. It was shown in mice models that treatments with vitamin K antagonists resulted in increased VC, both in mice with chronic kidney disease (CKD) and in those with normal renal function [3,4]. This also holds true for humans: the patients that are treated with warfarin are facing an increased VC compared to those who are not treated with vitamin K antagonists [5,6]. CKD patients are prone to developing VCs because they have a defective uremic and hyperphosphatemic balance [7]. Medial artery calcifications that seem to be more related to mineral metabolism changes have been considered separately from intimal calcifications of common atherosclerosis [8]. 

Warfarin has been prescribed in dialysis patients for a long time and continues to be so, as there is not enough data about new oral anticogulants in CKD [9]. Additionally, Vitamin K deficiency–related clinical consequences and their treatments have been investigated in recent years [10,11]. Although the evidences are circumstantial since the number of patients using warfarin is relatively low, and there are other mechanisms causing vascular calcifications in HD patients; warfarin and VC have nonetheless been known to be related [12,13]. The use of plain radiography of the abdominal aorta (Kauppila score) and/or the hands and pelvis (Adragao score) to rate vascular calcifications has been validated by various studies both in dialysis and predialysis patients. These studies also have a good correlation with coronary calcifications and mortality [14–16]. 

The aim of this study is to investigate the relationship between VC and warfarin use in hemodialysis patients and to emphasize the ease of revealing a VC with a simple method in order to prevent it from progressing further. 

## 2. Materials and methods

This is a cross-sectional, observational, case-control study of prevalent hemodialysis (HD) patients from six centers. This study was approved by Ankara University School of Medicine Ethics Committee for Clinical Studies (Approval Number: 10-798-19), and all the procedures were conducted in accordance with the Declaration of Helsinki. From the six hemodialysis centers, we included adult patients with their informed consent who had been on dialysis for >1 year. The patients who had been on warfarin at least 1 year were placed in study group. Out of a total 711 hemodialysis patients, there were 32 (4.5%) who had been treated with warfarin and we included, in the study, 44 random control HD patients with matching parameters of age, sex, comorbidities and dialysis vintage from the same centers as the aforementioned warfarin–treated patients (Table 1). After specifying the possible matched control patients in the same center and same dialysis session, the responsible doctors of each center randomly determined one or two control patients. Power analysis showed that a minimum sample size of 52 achieves 95% power to detect an effect size of 0.5 with a significance level of 0.05. In total, we included 76 patients to the study. All 76 patients had been treated with three-times-a-week hifg-flux HD and warfarin-treated patients had been receiving warfarin for at least 1 year. 

**Table 1:** Basic characteristics of the patients and summary of the results.

Parameter	Warfarin (+) n = 32	Warfarin (-) n = 44	p value
Age (years, mean±SD)	67.6 ± 9	64.54 ± 8	0.143
Female/Male	14, 43% / 18; 56%	22, 50% / 22, 50%	0.646
Diabetes mellitus +/- (n,%)	6, 18%	12, %27	0.427
Dialysis vintage (months, mean ± SD)	74.9 ± 44	63.6 ± 33	0.209
Kt/V	1.62 ± 0.2	1.7 ± 0.2	0.205
Warfarin usage time (months, mean±SD)	65.9 ± 43	NA	NA
Kauppila score (median, min-max)	11 (1-24)	6.5 (1-20)	0.032
Kauppila score > 6 (n, %)	23, 76.6%	22, 50%	0.029
Adragao score (median, min-max)	7 (2-8)	6 (0-8)	0.177
Adragao score ≥ 3 (n, %)	30, 93.7%	41, 93.1%	0.638
Vascular access· Fistula (n,%)· Central vein catheter (n,%)	23, 71.9%9, 28.1%	43, 97%1, 3%	0.001
Medical treatments (n,%)· Ca-containing P-binder· Non-Ca-containing P-binder· Calcimimetics· Active vitamin D· IV iron· ESA· Antiaggregant	13, 40%15, 46%5, 15%20, 62%22, 68%21, 65%6, 18%	14, 31%26, 59%14, 31%29, 65%31, 70%30, 68%26, 59%	0.4730.3540.1790.8111.01.0<0.001
Systolic blood pressure (mean±SD) (mmHg)	115.9 ±16.4	120.7 ± 23.1	0.319
Diastolic blood pressure (mean±SD) (mmHg)	67.5 ± 7.47	68.9 ± 12.69	0.540
Residual renal function (n, %)	13, 40%	14, 31%	0.473
Dialysate calcium ≥ 1.50 mmol/L (n,%)	14, 43.7%	15, 31.8%	0.475
Serum calcium (mg/dL) (mean±SD)	8.82 ± 0.5	8.78 ± 0.5	0.778
Serum phosphorus (mg/dL) (mean±SD)	4.62 ± 0.87	4.84 ± 0.89	0.279
Serum parathyroid hormone (pg/mL) (mean±SD)	376.23 ± 192.8	457.36 ± 251.2	0.116
Serum albumin (g/dL) (mean±SD)	3.81 ± 0.38	3.91 ± 0.52	0.361
Serum hemoglobin (g/dL) (mean±SD)	11.43 ± 1.1	11.6 ± 1.57	0.595
Serum C-reactive protein (mg/L) (mean±SD)	14.3 ± 12.2	13.8 ± 15.4	0.881
Serum LDL cholesterol (mg/dL) (mean± SD)	106.8 ± 41	107 ± 34	0.966
Serum HDL cholesterol (mg/dL) (mean± SD)	37.1 ± 10	38.9 ± 7	0.384
Serum triglyceride (mg/dL) (mean±SD)	158.9 ± 77	175.7 ± 89	0.374

Abbreviations: Ca, Calcium; ESA, erythrocyte stimulant agents; HDL, high density protein; IV, intravenous; Kt/V, K:dialyzer clearance of urea, t: dialysis time, V: volume of distribution of urea, approximately equal to patient’s total body water; LDL, low density protein; P, phosphorus; SD, standart deviation.

**Figure 1:**
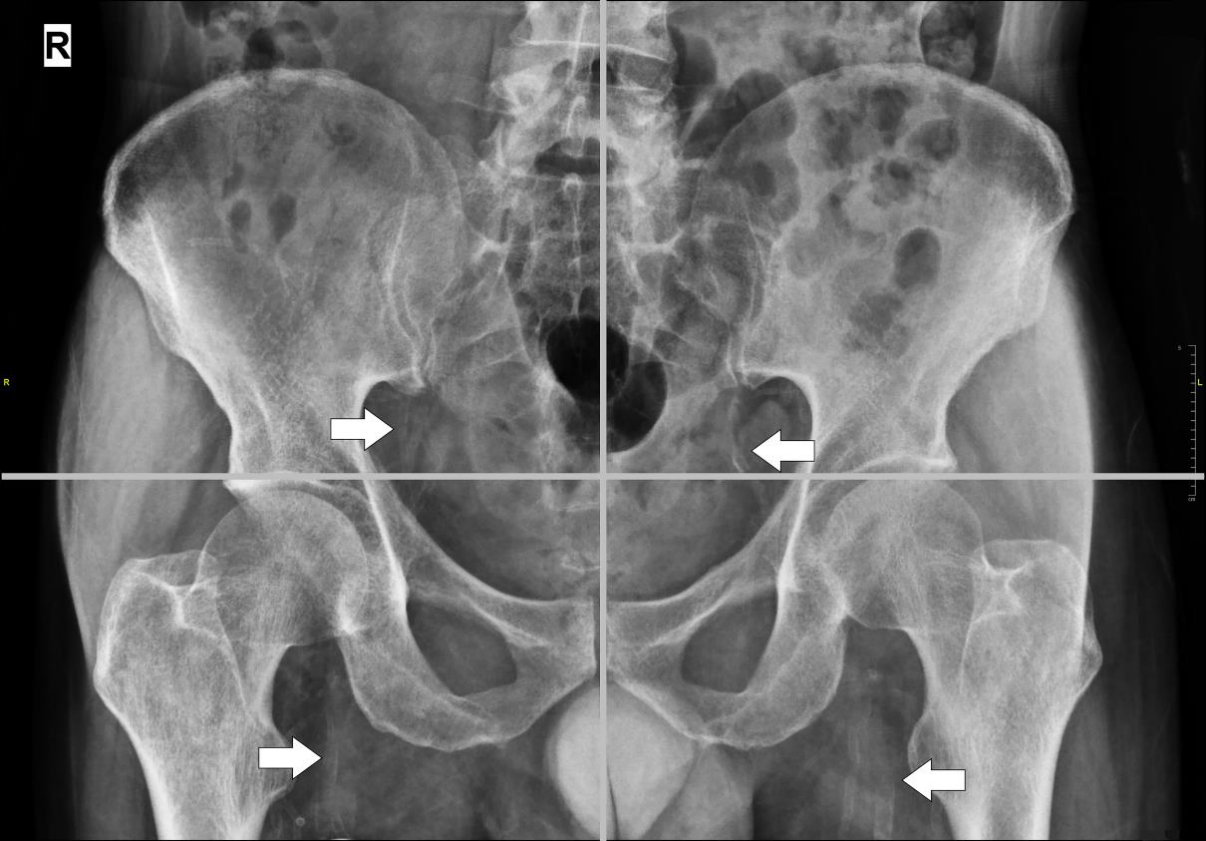
Pelvis graphy- iliac and femoral arterial calcifications.

**Figure 2:**
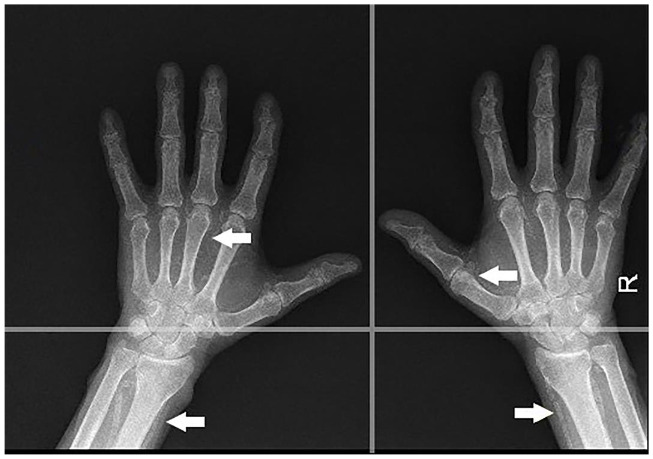
Hand graphy- radial and digital arterial calcifications.

Lateral abdominal radiography (includes all lumbar regions of the spinal column), frontal pelvic radiography, and radiography of both hands were done in July 2019. Femoral, iliac, digital and radial artery, and aortic vascular calcifications were assessed using Adragao (AS; pelvis and hands; min-max: 0-8) and Kauppila (KS; lateral lumbar spine; min-max: 0-24) scores outlined below as described in the literature. Pelvis films were divided into four sections by a horizontal line over the upper limit of both femoral heads and a median vertical line over the vertebral column. (Figure 1.) A horizontal line divided the films of each hand over the upper limit of the metacarpal bones. (Figure 2.) The presence of vascular calcifications in each section was rated as 1, and its absence as 0. Only linear calcifications that outline the vessel wall were considered as VC.

**Figure 3:**
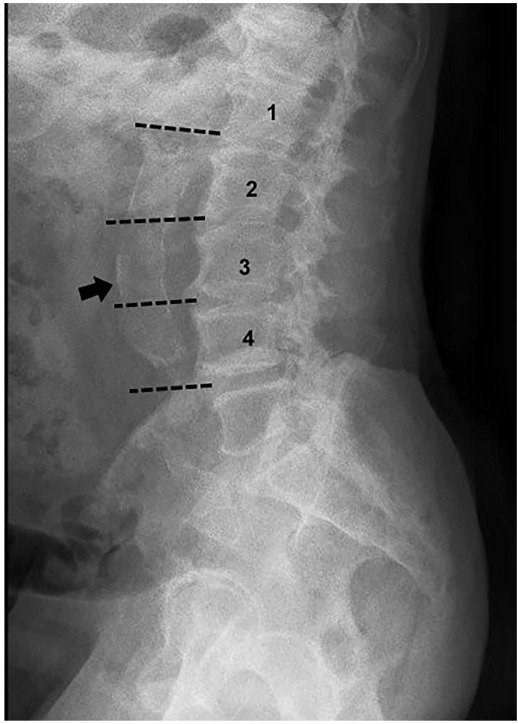
Lateral abdominal graphy- aortic calcifications.

The lateral abdominal radiographs were assessed separately for each lumbar vertebra for the posterior and anterior wall of the aorta using the midpoint of the intervertebral space above and below the vertebrae as the boundaries. (Figure 3.) The presence of small, scattered calcific deposits was rated 1 if they filled less than one-third of the longitudinal wall of the aorta; 2 if they filled less than two-thirds of; 3 if they filled two-thirds or more of it, and 0 if they were absent [16,17]. One single experienced radiologist blinded to patient information performed the analysis of all the radiographic films. 

Data regarding the demographic characteristics, duration of dialysis, duration of warfarin usage, dialysis adequacy parameters including Kt/V (K:dialyzer clearance of urea, t: dialysis time, V: volume of distribution of urea, approximately equal to patient’s total body water) and urea reduction ratio (URR), dialysis vascular access, blood pressure measurement records, residual renal function (intradialytic urine output >300 mL/day), concomitant treatments, laboratory results, and possible risk factors related to VC were recorded based on the records in 6 months preceding vascular calcification score evaluation. Calcium, phosphorus, hemoglobin, albumin, URR, and Kt/V were evaluated every month and serum PTH and C-reactive protein every 3 months. Blood pressure (BP) measurements were recorded before and after the mid-week dialysis session, during the same 6 months period. 

### 2.1. Statistical analysis

Clinical and laboratory data are expressed as percentages, means (± SD) or medians [interquartile range (IQR)], as appropriate. Continuous variables in the characteristics of the two groups were compared by t-test or Mann–Whitney U test, and categorical variables with Pearson’s chi-square or Fischer exact tests. Logistic regression analyses were performed to study associations between vascular calcification scores (dependent variable) and predictor variables. Parameters with a <0.2 p-value in univariate analysis were considered for entry in the multiple logistic regression model along with the clinical-related parameters. The final model was reached by backward stepwise regression (Backward LR) method incorporating constant. The quality of adjustment of the model was tested with the Hosmer–Lemeshow statistic. Odds ratios were expressed with 95% confidence intervals (CI). A threshold value of p<0.05 was considered as statistically significant. The calculations were made with IBM SPSS 23  (IBM SPSS v.23, Armonk, NY, USA).

## 3. Results

The average age of the participants was 66 ± 9 years, and 47% of them were females. Of the patients, 23% were diabetics, the mean dialysis vintage was 68 ± 38 months, and the mean Kt/V 1.66 ± 0.27. Out of a total of 76 patients, while 45 (60.8%) had an increased Kauppila score (median 8, min 1- max 24); 71 (94.7%) had an increased Adragao score (median 6, min 0- max 8). 

In the study, we included patients who had been taking warfarin for at least one year. Their mean duration of warfarin treatment was 65.9 ± 43 months, and median weekly dosage of warfarin was 25 mg (min 7.5 mg- max 40 mg). Warfarin indications were atrial fibrillation (n = 16), heart valve replacement (n = 9), recurrent pulmonary thromboembolism (n = 5), and recurrent deep venous thromboembolism (n = 2). 

In warfarin group, median Kauppila score was higher than in control group [11 vs 6.5, p = 0.032] and the percentages of the patients with a Kauppila score of >6 was higher, as well (76,6% vs. 50%; p = 0.029). Median Adragao score was not significantly different between the two groups [7 vs. 6, p = 0.177] (Table 1).

There were no significant differences detected in the clinical characteristics and basic laboratory results between control and warfarin group, except for vascular access (Table 1). In warfarin group, there were 9 patients (28.1%) with a central venous catheter, while there was only 1 such patient (3%) in control group. Considering the possible effects on vascular calcification, concomitant medications, including calcium-containing and noncalcium-containing phosphate binders, calcimimetics, vitamin D, dialysate calcium concentrations were recorded. There was no significant difference in the percentage of patients who have taken these medications. The only detail was that there were fewer patients taking antiaggregant agents such as aspirin or clopidogrel in warfarin group. There was also no difference in the basic laboratory results between the two groups.

To determine the independent risk factors for vascular calcification, we conducted logistic regression analysis. Age, warfarin treatment, serum parathormone, serum calcium, serum phosphorus, dialysis vintage were considered as possible independent risk factors for the reason that their p-values were <0.20 in univariate analysis or for their clinical relevance of vascular calcification. We conducted a multivariate analysis with these 6 possible variables and at last step of logistic regression analysis model, it was revealed that only the warfarin treatment was associated with Kauppila scores of >6 (OR 3.28, 95% CI 1.17-9.22, p = 0.024) (Table 2). Although Adragao scores were not different between warfarin and control groups, we checked for the same risk factors for an Adragao score of ≥3; however, we were not able to find a significant relation. 

**Table 2:** Multivariate analysis results for the risk factors for Kauppila score >6.

	Univariate		Multivariate *	
Parameter	p-value	Odds ratio (95% CI)	P value	Odds ratio (95% CI)
Warfarin	0.024	3.28 (1.17–9.22)	0.024	3.60 (1.18–10.97)
Age	0.192	1.03 (0.98–1.09)	NS	
Serum parathormone level (pg/mL)	0.651	1 (0.99–1.0)	NS	
Serum calcium level (mg/dL)	0.961	1.02 (0.41–2.49)	NS	
Serum phosphorus level (mg/dL)	0.964	0.988 (0.58–1.66)	NS	
Dialysis vintage (months)	0.785	1.00 (0.99–1.01)	NS	

Abbreviations: CI, confidence interval; NS, not significant.

## 4. Discussion

This study revealed that vascular calcification is observed in almost all dialysis patients, and that warfarin is a strong risk factor for vascular calcifications, especially in aorta of hemodialysis patients. We found that the presence of VC assessed by plain radiographies of the abdominal aorta is more frequent and severe probably because of the deleterious effects of warfarin on the vessels in patients who have been receiving warfarin for over a year. This is a direct study with relatively sufficient number of patients using warfarin, and it reveals that warfarin might accelerates the vascular calcifications in HD patients.

The mechanism of increased vascular calcification as a result of warfarin is presumably related to insufficient activation of MGP. There is evidence that the inactive form of MGP reflects vitamin K-deficiency as a result of warfarin treatment, and this is associated with medial arterial calcification in HD patients [18]. In addition, they showed decreased dephosphorylated-undercarboxylated MGP (dp-ucMGP) after Vitamin K supplementation [19]. Tantisattamo et al. compared the screening mammograms in patients who had been treated with and without warfarin and showed that, even in non-renal population, warfarin treatment is associated with increased VC [4]. Additionally, they indicated that the prevalence of VC increased even more with a longer treatment duration, especially >6 months. Arterial wall properties change with advanced age; however, the prevalence of VC increases with the advancing stage of CKD even in young patients in whom medial calcification was found to be more prevalent than intimal calcification and to be related to cardiovascular mortality [20,21]. To avoid such a conflict, we only included the patients who had been receiving warfarin for at least 1 year, and we compared them with the patients matching for age, sex, diabetes mellitus, and dialysis vintage.

The diagnosis of vascular calcification has been made with expensive and technical devices such as multislice computed tomography [22]; however, plain radiographic films are practical, inexpensive, and they correlate with advanced techniques [23]. Plain films are also suggested in the KDIGO 2017 guidelines for the assessment of VC in CKD [24]. Fusaro et al. [13] used lumbar graphies to reveal aortic and iliac VC with a different assessment method which interprets in centimeters the entirety of the calcifications occuring only on one edge of the vessel [25], and they still found the same relation between VC and warfarin. Recently, the Valkyrie Study [12], a randomized controlled trial, has tested the effect of warfarin, rivaroxaban and vitamin K2 supplementation on VC progression detected by computed tomography of the heart and thoracic aorta in hemodialysis patients with atrial fibrillation. They did not find a difference in the VC progression despite a significant decrease in dp-ucMGP levels. Their study population consisted mostly of prevalent hemodialysis patients who had been taking warfarin at the inclusion of the study, and they only evaluated the thoracic part of the aorta for VC.

The presence of VC may be heterogeneous, such as classical atherosclerosis in the context of aorta; medial calcification, in the context of muscular arteries, cardiac valve calcifications; and calciphylaxis. While common atherosclerotic process is linked to inflammation, dyslipidemia or advance age, medial calcification seems to be more related to bone-mineral disorders or CKD. Mourad et al. [26] tried to prove this phenomenon by measuring the elevated stiffness and the diameter of radial arteries of end-stage renal disease (ESRD) patients and comparing them to control groups. They indicated that muscular arterial wall alterations should be related to the ESRD status, and not to blood pressure changes.

Adragao et al. [15] identified a simple score evaluating iliac, femoral, radial and digital arteries, and they found that the cut-off value of ≥3 is the best association with cardiovascular morbidity and mortality. Kauppila et al. [16] developed a score of calcification of abdominal aorta based on the lateral abdominal films obtained from Framingham heart study participants, and prominent VC was scored as >6. Elevated Kauppila score is also shown to be related to mortality in hemodialysis patients [27]. Gorriz et al. [14] used both scores in their study with non-dialysis CKD patients, and they found an Adragao score of ≥3 (but not a Kauppila score of >6) that is independently associated with all-cause and cardiovascular mortality. Additionally, age, phosphorus, and diabetes were found to be related to Adragao score, which is consistent with Mourad’s aforementioned study [26]. 

We used the same validated scores because they are very simple and easy to use and may help distinguish the high risk patients earlier. In our study, the percentages of the patients with an Adragao score of ≥3 were 93.7% and 93.1%, and those with a Kauppila score of >6 were 76.6% and 50% in warfarin and control groups, respectively. It is probably related to the capacity of Kauppila score that allows it to show calcifications in a wider range of intensity as a Kauppila score would rate a given region as 0–3, as opposed to an Adragao score, which would rate it as 0–1 for an artery. We found that increased aortic calcification is associated with warfarin usage; however, we could not show the difference in peripheral and iliofemoral arteries (Adragao scores) because it had already increased in almost all the patients in both groups.

Although hyperphosphatemia has been associated with VC [28], Adragao et al. [17] found no correlation between total VC scores and calcium, phosphate or PTH levels in their study. Choi et al. [29] concluded that hypoalbuminemia, high CRP, low LDL-cholesterol, and the presence of baseline calcification in aorta were found to be significant risk factors for the progression of abdominal aortic calcification, which was assessed by lateral abdominal radiography. In some studies, it is suggested that cinacalcet might reduce vascular calcification [30]. In our study, however, there was no difference between the two groups in terms of concomitant drugs, electrolytes, PTH, CRP, anemia or lipid status. Additionally, in logistic regression analysis, we took into account the age, warfarin treatment, serum parathormone, serum calcium, serum phosphorus, and dialysis vintage as risk factors and only warfarin was independently associated with abdominal aortic calcifications. 

There is conflicted data regarding the benefit of using warfarin in dialysis patients in order to prevent cerebrovascular events in patients with atrial fibrillation [31]. Given the fact that these patients are more prone to cardiovascular diseases, it seems prudent to restrict the wide prescription of warfarin at least for atrial fibrillation in dialysis patients. Recently, apixaban has been approved for use in patients with atrial fibrillation and ESRD in USA [32], and it may become a solution to this conflict.

Our study has some limitations. The small number of patients may influence the statistical significances. Although we had a matching control group, we did not have the chance to assess initial Adragao and Kauppila scores at the beginning of warfarin treatment. In terms of matching differences, antiaggregant agent usage was lower in warfarin group; this probably reflected the intention of preventing further bleeding complications. The cross-sectional nature of our study did not allow the identification of all the factors associated with VC progression and/or mortality. We did not measure the dp-ucMGP levels, which is an accurate biomarker for vascular vitamin K stores. 

In conclusion, based on our results, long-term warfarin treatment is associated with vascular calcification, and it should be used with extreme caution in hemodialysis patients.

## Informed consent

This study was approved by Ankara University School of Medicine Ethics Committee for Clinical Studies (Approval Number: 10-798-19), and all the procedures were conducted in accordance with the Declaration of Helsinki. Written informed consents expressing propriate statements related to participation and publication were obtained from all the patients prior to the study.
